# Nonlinear control of photonic higher-order topological bound states in the continuum

**DOI:** 10.1038/s41377-021-00607-5

**Published:** 2021-08-10

**Authors:** Zhichan Hu, Domenico Bongiovanni, Dario Jukić, Ema Jajtić, Shiqi Xia, Daohong Song, Jingjun Xu, Roberto Morandotti, Hrvoje Buljan, Zhigang Chen

**Affiliations:** 1 The MOE Key Laboratory of Weak-Light Nonlinear Photonics, TEDA Applied Physics Institute and School of Physics, Nankai University, 300457 Tianjin, China; 2 INRS-EMT, 1650 Boulevard Lionel-Boulet, Varennes, QC J3X 1S2 Canada; 3 Faculty of Civil Engineering, University of Zagreb, A. Kačića Miošića 26, 10000 Zagreb, Croatia; 4 Department of Physics, Faculty of Science, University of Zagreb, Bijenička c. 32, 10000 Zagreb, Croatia; 5 Collaborative Innovation Center of Extreme Optics, Shanxi University, 030006 Taiyuan, Shanxi China; 6 Institute of Fundamental and Frontier Sciences, University of Electronic Science and Technology of China, 610054 Chengdu, Sichuan China; 7 Department of Physics and Astronomy, San Francisco State University, San Francisco, CA 94132 USA

**Keywords:** Micro-optics, Nonlinear optics, Other photonics

## Abstract

Higher-order topological insulators (HOTIs) are recently discovered topological phases, possessing symmetry-protected corner states with fractional charges. An unexpected connection between these states and the seemingly unrelated phenomenon of bound states in the continuum (BICs) was recently unveiled. When nonlinearity is added to the HOTI system, a number of fundamentally important questions arise. For example, how does nonlinearity couple higher-order topological BICs with the rest of the system, including continuum states? In fact, thus far BICs in nonlinear HOTIs have remained unexplored. Here we unveil the interplay of nonlinearity, higher-order topology, and BICs in a photonic platform. We observe topological corner states that are also BICs in a laser-written second-order topological lattice and further demonstrate their nonlinear coupling with edge (but not bulk) modes under the proper action of both self-focusing and defocusing nonlinearities. Theoretically, we calculate the eigenvalue spectrum and analog of the Zak phase in the nonlinear regime, illustrating that a topological BIC can be actively tuned by nonlinearity in such a photonic HOTI. Our studies are applicable to other nonlinear HOTI systems, with promising applications in emerging topology-driven devices.

## Introduction

Over the past decade, topological insulators have attracted tremendous attention across many disciplines of natural sciences^1,2^, including photonics^3^. One of their most appealing features is the topologically protected edge states immune to scattering at defects and disorder^4–6^. A few years ago, a novel class of higher-order topological insulators (HOTIs) was predicted^7–11^ and experimentally observed^12–14^. While a traditional topological insulator usually obeys the bulk–boundary correspondence principle (with edge states one dimension lower than the bulk), HOTIs typically support zero-dimensional corner states regardless of their physical dimension, or more generally, (*d*–*n*)-dimensional states at the boundaries of *d-*dimensional lattices with *n* no less than 2^3,15^. This is associated with topologically quantized quadrupole and higher-order electric moments in electronic systems^7^. The discovery of HOTIs broadened the concept of the symmetry-protected topological phase and our common understanding of traditional topological insulators, which has thus launched a host of research ventures on HOTIs in a variety of fields, including condensed matter physics, electric circuits, acoustics, and photonics^7,10,12–14,16–32^. In terms of fundamental interest, HOTIs are attractive because they are related to many intriguing phenomena, such as higher-order band topology in twisted Moiré superlattices^33^, topological lattice disclinations^34^, and Majorana bound states^35^ and their nontrivial braiding^36^. Toward applications, they have been touted and tested for robust photonic crystal nanocavities^37^ and low-threshold topological corner state lasing^29,38^.

Combining topology and nonlinearity leads to a number of fundamental questions, some of which have been addressed in the study of first-order nonlinear topological photonic systems^39^, including, for example, nonlinear topological solitons and edge states, nonlinearity-induced topological phase transitions, topological nonlinear frequency conversion, and nonlinear tuning of non-Hermitian topological states^40–47^. However, thus far, all of the studies on HOTIs have mainly been restricted to the linear regime, and only recently it became clear that unexpected phenomena may arise when nonlinearity is taken into account in HOTI systems^48–50^, with experiments implemented already in nonlinear electric circuits^48^ and photonic structures^51–53^.

In a few recent papers, an intriguing connection between HOTIs and another widely studied phenomenon, namely, the bound states in the continuum (BICs), has been explored^22,54,55^, reinvigorating the interest in BICs and their topological nature, as previously established^56–58^. BICs are counter-intuitive localized states with eigenvalues in the continuum of extended states, which may result from versatile mechanisms^57–60^. An exemplary model used for unveiling such a connection is the celebrated two-dimensional (2D) Su–Schrieffer–Heeger (SSH) lattice^61^, which possesses the second-order localized modes protected by both chiral symmetry and crystalline symmetry^54^, as observed in a variety of synthetic structures^12–14^ including photonic crystals^18,19^. In such HOTIs, the corner-localized states appear right at the center of the eigenvalue spectrum (zero-energy mode) and are embedded in the continuum rather than in the gap, in contrast to other types of HOTIs^20,24,62^. Such topological BICs have infinite lifetimes and are fully localized to the corner despite being embedded in the bulk band, but they become “leaky” when the required symmetries are broken^54,55^. Nonlinearity can be used to break these symmetries and thus enable coupling of light into/out of these localized corner states or facilitating their interaction, therefore making them attractive for potential applications. However, to the best of our knowledge, nonlinear higher-order BICs and their associated dynamics have not been explored so far in photonics or any other systems. (Although a recent work^63^ reported nonlinear second-order photonic topological insulators, the presence of topologically nontrivial corner-localized states in crystalline insulators based on the breathing Kagome lattices has become a topic of debate. Furthermore, corner-localized higher-order BICs do not exist in those systems.)

**Fig. 1:**
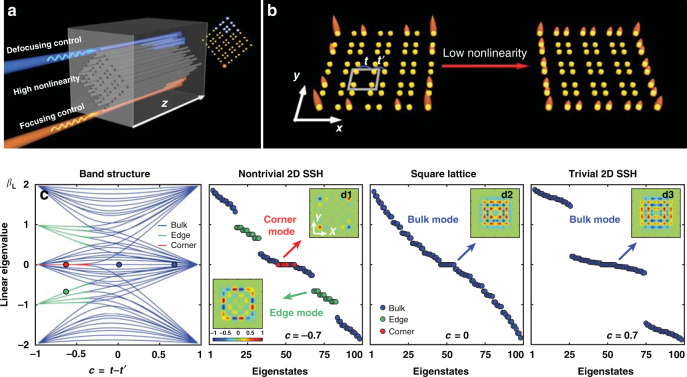
Illustration of nonlinear control of a higher-order topological insulator. **a** Schematic of corner excitations in a 2D SSH photonic lattice under high nonlinearity, where a focusing nonlinearity leads to corner soliton formation while a defocusing nonlinearity leads to radiation into the bulk/edge. **b** Illustration of coupling and beating between corner and edge states under weak nonlinearity. **c** Calculated *linear* eigenvalues of the SSH lattice *β*_L_ as a function of the dimerization parameter *c*, where the corner and edge states are highlighted with red and green colors in the highly topologically nontrivial regime. **d1**–**d3** Calculated band structures for the nontrivial, square, and trivial lattices, showing the topological phase transition as the dimerization parameter is tuned, where the insets plot the selected mode profiles corresponding to the marked color points. A topological BIC with a characteristic corner-localized mode profile is shown in the upper-right inset of **d1**, with zero amplitude in the nearest neighboring sites but nonzero amplitude and opposite phase in the NNN sites

Here we establish a nonlinear photonic HOTI platform and explore the role of nonlinearity in higher-order topological BICs (Fig. 1a, b). We demonstrate that a low nonlinearity, either self-focusing or self-defocusing, can induce coupling between corner states and edge states in a 2D SSH nontrivial lattice, enabling their beating oscillations. Nonlinearity can break the symmetries in the lattice. Consequently, an excitation of the corner-localized BICs is expected to radiate into the bulk. Yet we show that, counter-intuitively, the excitation of the corners leads to the beating between the corner BICs and the edge modes with appropriate nonlinearity strengths rather than coupling to the bulk modes. Surprisingly, even a self-defocusing nonlinearity does not favor the coupling with the bulk modes. Only at a high nonlinearity, a corner state can become more localized and form a semi-infinite gap soliton out of the continuum, or it can exhibit strong radiation into the edge and bulk. We theoretically analyze the dynamical evolution of the nonlinear eigenvalue spectrum and validate the robustness of the corner modes in the process of beating with the edge modes driven by a low nonlinearity. This is supported by calculating the bulk polarizations manifesting the topological invariant in the nonlinear regime as well as by simulating long-distance beam propagation. Our work not only provides another direct experimental evidence of the topological BICs recently predicted in linear systems^54^ but also, more importantly, unveils the intriguing nonlinear dynamics of topological BICs in HOTIs.

## Results

The wave dynamics in our HOTI system can be described by the *continuous* nonlinear Schrödinger-like equation (NLSE), typically used for simulating a light field with amplitude *ψ*(*x, y, z*) propagating along the longitudinal *z*-direction of the photorefractive photonic lattice^64^:

1 $$i\frac{{\partial \psi }}{{\partial z}} + \frac{1}{{2k}}\left( {\frac{{\partial ^2\psi }}{{\partial x^2}} + \frac{{\partial ^2\psi }}{{\partial y^2}}} \right) - \Delta n\frac{\psi }{{1 + I_{\rm{L}} + I_{\rm{P}}}} = 0$$

where *I*_L_(*x, y*) accounts for the beam intensity required for writing the 2D SSH lattice in a nonlinear photorefractive crystal, *I*_P_ is the nonlinear contribution of the probe beam that is proportional to |*ψ*(*x, y, z*)|^2^, *k* is the wavenumber in the crystal, and Δ*n* is the linear refractive index change determined by the bias field *E*_0_ and by the electro-optic coefficient of the crystal (see Supplementary Material). The induced refractive index change forming the linear photonic lattice depends on the spatial coordinates *x* and *y*, and it is uniform along the propagation axis as illustrated in Fig. 1a. The nonlinearity can be self-focusing or self-defocusing, depending on the direction of the bias field relative to the crystalline optical axis, while its strength can be controlled by the bias field and the beam intensity^64,65^. It should be pointed out that, for low power probe beams (small *I*_P_), the nonlinearity is in the low saturable regime and approximately Kerr-like, then the above NLSE is equivalent to the Gross–Pitaevskii equation that describes interacting atomic Bose–Einstein condensates in the mean-field approximation^2^. As such, even though we used a specific type of optical nonlinearity in our study, the concept and scheme of nonlinear control of HOTI corner modes developed here are expected to hold in other platforms beyond photonics.

The topological features of the 2D SSH lattice are more transparent in the discrete model based on the coupled-mode theory. When the next-nearest-neighbor (NNN) coupling is negligible, Eq. (1) can be approximated with

2 $$i\frac{{\partial \psi _\alpha }}{{\partial Z}} + \mathop {\sum }\limits_\alpha \left[ {H_{\rm{L}}} \right]_{\alpha ,\alpha ^{\prime}}\psi _{\alpha ^{\prime}} + E_0^\prime \frac{{\gamma \left| {\psi _\alpha } \right|^2}}{{1 + \gamma \left| {\psi _\alpha } \right|^2}}\psi _\alpha = 0$$

where *ψ*_*α*_ is the complex amplitude of the electric field at the site *α*, *H*_L_ is the linear Hamiltonian matrix of the 2D SSH model; its entries [*H*_L_]_*α*__,*α*′_ are either zero (when *α* and *α*′ are not neighboring sites), or take the value of either the intracell coupling *t* or the intercell coupling *t*′, as illustrated in Fig. 1b. Both the normalized bias field ${E_{0}}^{\prime}$ and the nonlinear coefficient γ control the saturable nonlinearity, which corresponds to the nonlinear photorefractive crystal used in the experiment^64,65^.

In the linear regime, in full analogy with the one-dimensional case^66,67^, the 2D SSH lattices exhibit two distinct Zak phases, which correspond to bulk polarizations. These are the topological invariants for this type of HOTIs^18,19,61^, which differentiate the topologically trivial and nontrivial structures (see “Methods”). They can be tuned by the dimerization parameter *c* = *t* – *t*′. In the discrete model, the topological characteristics of the 2D SSH lattices can be clearly seen from the linear eigenvalue spectrum calculated from *H*_L_*φ*_L,*n*_ = –*β*_L,*n*_*φ*_L,*n*_, as summarized in Fig. 1c, d, where *β*_L,*n*_ is the linear spectrum and *φ*_L,*n*_ are the corresponding eigenmodes. When the intracell coupling is weaker than the intercell coupling (*c* < 0), the system is in the topologically nontrivial phase, and the band structure consists of characteristic edge and corner modes (Fig. 1c). In particular, in the middle of the band, there are four degenerated corner modes (Fig. 1d1), corresponding to “zero-energy modes” in the condensed matter language. A typical corner mode structure is shown in the upper inset of Fig. 1d1, which clearly displays the features of the topological corner state (highly localized at the corner with zero amplitude in its nearest-neighbor sites but nonzero out-of-phase amplitude in its NNN sites along the edges). Since these corner states are embedded in the continuum of the SSH lattice as well as protected by the *C*_4__*v*_ and chiral symmetries, they are topological BICs^54,55^. Their exponential localization characteristics are further analyzed with a larger topological lattice structure (see Supplementary Materials). For comparison, the trivial phase, manifested by vanishing polarizations, occurs when the intracell coupling is stronger than the intercell coupling (*c* > 0), leading to two mini-gaps formed only by the bulk modes (Fig. 1d3). When the coupling strength is uniform across the whole lattice (*c* = 0), it turns to a trivial square lattice with a gapless spectrum (Fig. 1d2), which sets apart the topologically nontrivial and trivial regimes. Representative edge and bulk modes are also displayed in the insets of Fig. 1d.

**Fig. 2:**
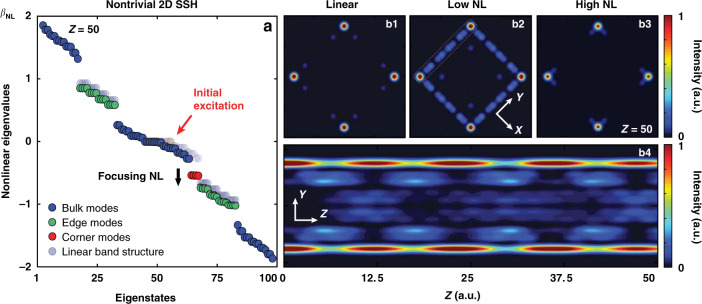
Calculated nonlinear band structure and corner mode tuning under a self-focusing nonlinearity. **a** Calculated *nonlinear* eigenvalues of the 2D SSH lattice *β*_NL_ for the nontrivial lattice using the discrete model, where the transparent dots are linear modes superimposed for direct comparison. The black arrow shows that four corner states (red dots) undergo coupling and beating with lower edge states under low self-focusing nonlinearity (see also Supplementary video), and the red arrow marks the initially excited corner mode that sustains the topological feature under linear conditions as shown in **b1** without any light distribution in the nearest neighboring sites. Under a low focusing nonlinearity, the corner mode couples with the edge modes (**b2**), and a beating oscillation occurs. This can be clearly seen from the side-view propagation of **b4**, taking from the upper-left edge marked by a dashed line in **b2**. Under a high focusing nonlinearity, a localized semi-infinite gap discrete soliton forms at the corners, with light distributing in the nearest neighboring sites (**b3**)

In the nonlinear regime, the eigenvalues are calculated from *H*_NL_*φ*_NL,*n*_ = −*β*_NL,*n*_(*Z*)*φ*_NL,*n*_(*Z*) where the nonlinear Hamiltonian *H*_NL_ = *H*_L_ + *V*_NL_ contains both the linear part and the nonlinear potential corresponding to the third term in Eq. (2). It is important to point out that the nonlinear eigenmodes *φ*_NL,*n*_(*Z*) and nonlinear eigenvalues *β*_NL,*n*_(*Z*) are *Z*-dependent (here *Z* is the normalized propagation distance playing the role of time) because the nonlinear beam dynamics are generally not stationary. Indeed, we use a general theoretical protocol (developed recently in ref. ^44^) for interpreting the dynamics in nonlinear topological systems, where both inherited and emergent topological phenomena may arise. The calculated nonlinear eigenvalue spectrum (at *Z* = 50) for the nontrivial SSH lattice is plotted in Fig. 2a, where two sets of edge modes set apart the whole band as in the linear spectrum (Fig. 1d1). However, under the action of nonlinearity, the spectrum exhibits a dynamical evolution during propagation, while the corner modes are forced to couple with the lower (upper) edge states by a self-focusing (self-defocusing) nonlinearity (see Supplementary Movies). In other words, they are no longer stationary BICs but rather undergo periodic energy exchange with the edge modes. The robustness of the corner localized BICs is evident, as they do not want to couple with the bulk modes even when they are driven in and out of the central bulk band via nonlinearity, reflecting the inherited topological nature of BICs. The detailed analysis of the intensity profile of these nonlinear corner modes on the logarithmic scale, along with their long-range propagation dynamics, can be found in the Supplementary Material. For the snapshot selected at *Z* = 50 (shown in Fig. 2a), the whole spectrum is down-shifted from its linear position by the focusing nonlinearity, while the corner modes are approaching and coupling with the lower edge modes. This shifting direction is reversed when a self-defocusing nonlinearity is employed. When the strength of nonlinearity is low (${E_{0}}^{\prime}$ = 5, *γ* = 1.1), a representative corner mode excited by the focusing nonlinearity undergoes beating with the edge modes (Fig. 2b2, b4), but only at a sufficiently high nonlinearity (${E_{0}}^{\prime}$ = 5, *γ* = 3.5) it is “liberated” from the continuum and turns into a self-trapped semi-infinite gap soliton (Fig. 2b3). Such corner solitons, which are formed only in the strongly nonlinear regime with eigenvalues (i.e., propagation constants) residing beyond the lattice Bloch band, have been explored previously in 2D square lattices^65,68^ but not in the context of HOTIs.

**Fig. 3:**
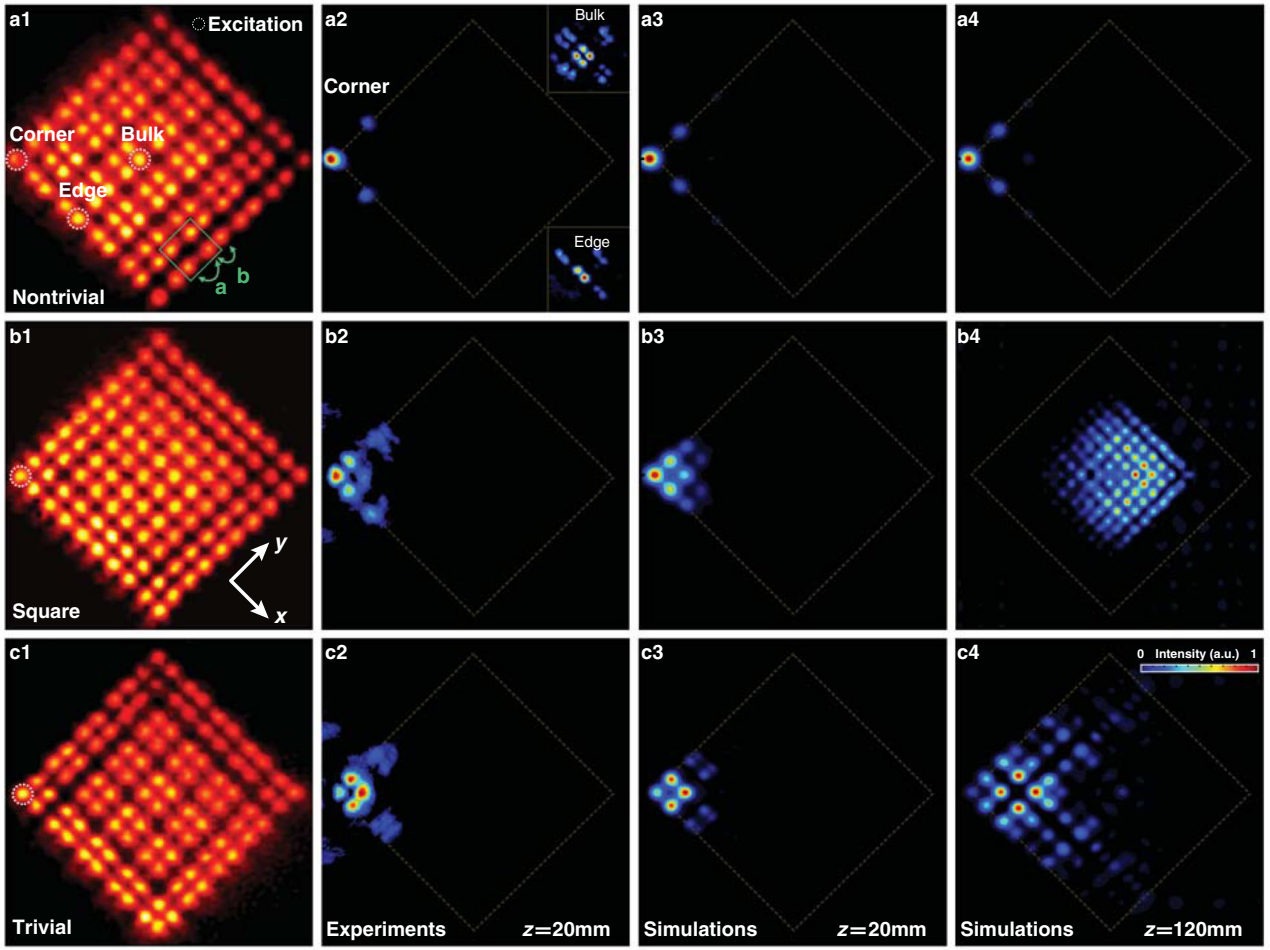
Experimental realization and probing of linear 2D photonic SSH lattices. **a1**–**c1** Laser-written 2D SSH lattices tuned to nontrivial, square, and trivial regimes at different dimerizations, where the dashed circles indicate the lattice sites for corner, edge, and bulk excitations, and *a* and *b* mark the waveguide distances for the weak and strong coupling bonds. **a2**–**c2** Experimental results for a linear output corresponding to single-site excitations in **a1**–**c1**, where the corner excitation in **a1** leads to a localized BIC with evident topological features: no light distribution appears in the nearest neighboring sites, but we observe a non-zero intensity in the NNN sites along two edges. Discrete diffraction is observed for all other excitations. **a3**–**c3**, **a4**–**c4** Numerical results corresponding to corner excitations in **a2**–**c2** obtained using the continuum model, where the propagation distance is **a3**–**c3** 20 mm corresponding to the length of the crystal used in the experiments and **a4**–**c4** 120 mm corresponding to a longer propagation, for direct comparison. Experimental parameters: *a* = 31 μm, *b* = 23 μm; the bias field during the writing process is *E*_0_ = 130 kV m^−1^

In our experiments, we establish the nonlinear photonic HOTI platform by site-to-site writing of the 2D SSH lattices in a photorefractive crystal, carried out with a continuous-wave (CW) laser^69^. Experimental details can be found in “Methods.” For direct comparison, the lattices are written into three different structures (nontrivial SSH, square, and trivial SSH, as illustrated in the left panels of Fig. 3) by tuning the dimerization parameter, in accordance with Fig. 1d1–d3, which in the experiment is achieved by controlling the intracell and intercell waveguide distances. We then excite the same corner site with a single Gaussian probe beam. Results obtained under *linear* excitation are shown in Fig. 3, where the probe beam itself has no nonlinear self-action but evolves into a characteristic corner state with a non-zero intensity distribution at the two NNN sites along the edges (Fig. 3a2), representing a typical topological BIC realized in the nontrivial SSH lattice. For all other cases of excitation, either at the edge and bulk of the nontrivial lattices or at the same corner of the trivial lattices, the probe beam is not localized but instead displays strong radiation into the bulk/edge as shown in Fig. 3a2–c2. To simulate such linear corner excitation, we set *I*_P_ = 0 in Eq. (1) and display the results obtained from numerical simulations for three different lattices in Fig. 3a3–c3, a4–c4, which agree well with our experimental observations.

**Fig. 4:**
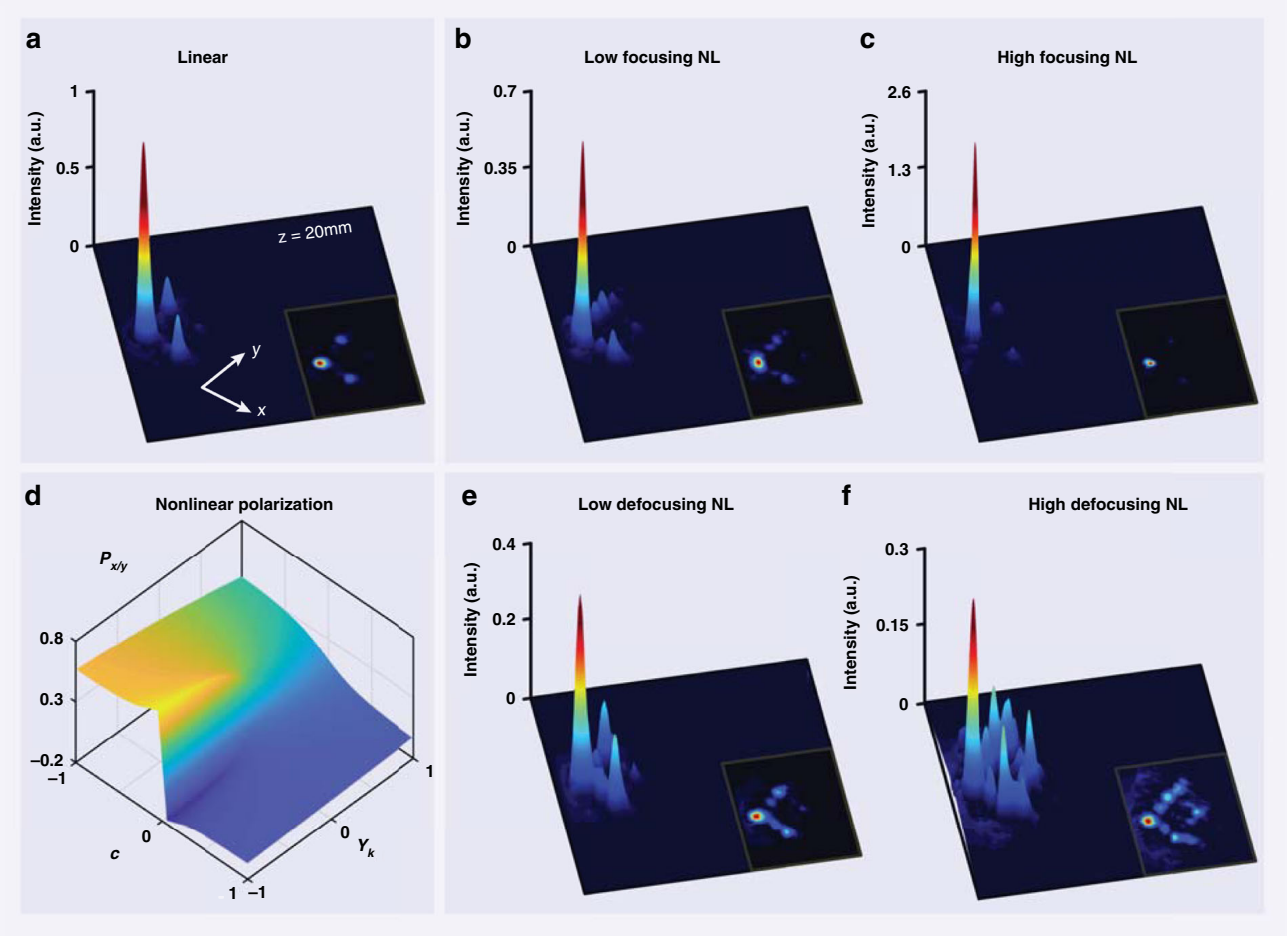
Experimental demonstration of the nonlinear control of a higher-order topological insulator. **a** 3D view of a typical linear corner state experimentally observed in a nontrivial lattice. **b**, **c** Nonlinear self-focusing leads to **b** coupling into the edges (non-zero intensity along the edge sites compared with the linear case) when the nonlinearity is low and **c** a highly localized corner soliton when the nonlinearity is high. **d** Plot of the calculated nonlinear polarization as a function of the nonlinear control parameter *γ*_*k*_ as well as the dimerization parameter *c*. Characteristic jump in the bulk polarization, testifying that the topological phase transition extends beyond the linear regime (*γ*_*k*_ = 0) because of the inherited topology in the nonlinear regime. **e**, **f** Experimental results of the nonlinear control with a low and high self-defocusing nonlinearity. Under a high defocusing nonlinearity, the energy spreads dramatically to both the edge and the bulk (**f**). For the focusing (defocusing) case, the bias field is *E*_0_ = 160 kV m^−1^ (*E*_0_ = −80 kV m^−1^), and the average power of the probe beam is about 15 nW (70 nW) for the low (high) nonlinearity. See Supplementary Material for the corresponding numerical simulation results

We now discuss the experimental results pertinent to the *nonlinear* control of HOTI corner states as illustrated in Fig. 1a, b and analyzed in Fig. 2. Measurements of the output intensity profile of the probe beam under a corner excitation of the nontrivial lattice with both self-focusing and self-defocusing nonlinearities after 20 mm propagation are shown in Fig. 4, where for reference Fig. 4a plots the linear output of the topological corner state. At a low nonlinearity, a direct comparison with the linear output shows that the corner-localized state differs in this case from the topological corner mode, since now the energy goes to the second (nearest neighbor) and even the fourth sites along the edges [Fig. 4b, e]. This is because of the nonlinearity-induced coupling between the corner and edge modes, as illustrated in Fig. 1b. In our experiments, because the propagation distance set by the crystal length is typically smaller than the beating period, we cannot observe the distinct beating oscillation between the corner and edge modes numerically shown in Fig. 2b4. At a high self-focusing nonlinearity, the probe beam is localized again in the corner, forming a self-trapped semi-infinite gap corner soliton as shown in Fig. 4c^65,68^, also in agreement with what is illustrated in Fig. 2b3. On the other hand, at a high defocusing nonlinearity, the corner excitation leads to strong spreading of the energy into the bulk as well as into the edges (Fig. 4f) due to nonlinear mode beating involving higher-band bulk states. These experimental results are corroborated by our numerical simulations based on the NLSE of Eq. (1) (see Supplementary Material for details), clearly demonstrating the concept of nonlinear control of the topological BICs using a photonic HOTI platform.

## Discussion

The concept of topologically protected (higher-order) BICs, which is explored here under the action of nonlinearity, is somewhat an oxymoron. If two states are close in energy (or, in the optics language, the propagation constants are close), then it should be easy to couple these states. In a nonlinear HOTI system, topology is involved in the dynamics, and thus for the system studied here this common sense is questioned.

In our theoretical simulations and experiments, we have clearly found that topological higher-order BICs, which are corner states of our nonlinear 2D SSH lattice, are dominantly coupling to edge rather than bulk states. This happens despite the fact that the corner-localized BICs are embedded in the continuum of bulk states, gapped from the edge states, as clearly illustrated in Fig. 1d1. A weak self-focusing or self-defocusing nonlinearity, for practically all excitations employed in this work, breaks the chiral symmetry and the crystalline symmetry of the lattice (the only exception is the excitation in Fig. 2, which preserves the *C*_4*v*_ symmetry). By breaking these symmetries, nonlinearity in principle allows the corner states to couple with the bulk states of the continuum in which they are embedded^54^. However, the overlap with the edge states induced by the nonlinearity is obviously much larger, which leads to the dominant coupling between the corner and the edge states. It should be noted that this behavior depends (also somewhat unintuitively) on the dimerization parameter *c*. If its magnitude increases (on the highly topologically nontrivial side), the gap between the topological corner BICs and the edge states increases (Fig. 1c), yet the dominant corner–edge coupling persists as opposed to the corner–bulk coupling.

A feature of interest for technological applications is the possibility to nonlinearly couple two corners; such coupling was proposed with exciton–polariton corner modes^49^. From the simulations presented in Fig. 2, it follows that such coupling should be possible in our lattice. Namely, if one excites a single corner, the initial state is then a superposition of four corner states that will beat; another view of the dynamics is that the nonlinearity will enable coupling to the edge states and then to the adjacent corner. This type of dynamics is verified in our numerical simulation under proper initial conditions. It should be noted that for our experiment the longest crystal we can possibly obtain has a length of only 20 mm, which corresponds to only a few coupling lengths between adjacent waveguides, a much smaller value than the beating oscillation period (around 200 mm along the propagation direction—see Fig. S4 in Supplementary Material). Therefore, it is not possible to experimentally observe the beating dynamics presented in Fig. 2 within the length of the nonlinear crystal. However, by using longer samples with different lengths or using different platforms with stronger coupling, such beating oscillations should be observed.

The distinction between the discrete and the continuous models under large nonlinearities merits further discussion. The continuous NLSE of Eq. (1) offers a quantitatively better description of the experiment than the discrete model in Eq. (2) under the tight-binding approximation. However, it is well known when the two models start to deviate. For a linear lattice that is sufficiently deep, the discrete model is a good approximation of the dynamics; the parameters of the linear lattice employed here are in this regime. When the nonlinearity is weak, the lattice will not be strongly perturbed, and the discrete model is still a good approximation. However, for a large self-defocusing nonlinearity, the whole lattice structure at the excitation can be strongly deformed. For example, if a corner gets excited, a large self-defocusing nonlinearity significantly molds the corner area and enables coupling between the NNN sites and changes the nearest-neighbor coupling as well, which is not captured by the discrete model of Eq. (2). In contrast, for a large self-focusing nonlinearity, the whole lattice structure is preserved despite the deep potential at the excitation site. In particular, we found that the discrete model is still qualitatively accurate for the presented self-focusing dynamics.

Quite generally, for a weak nonlinearity and practically any excitation, the symmetries responsible for the nontrivial topology of the 2D SSH model are broken^54^. However, in a weakly nonlinear system, the topological properties can persist as they are *inherited* from the corresponding linear system^44^. This is the origin of the weak nonlinear coupling between the corner and the bulk modes discussed above. The fact that the topological properties are inherited is quantified and illustrated in Fig. 4d, showing the bulk polarizations *P*_*x*_ and *P*_*y*_ (related to the 2D Zak phase^16,18^) as a function of the parameter *c* and the strength of the nonlinearity *γ*_*k*_. The nonlinear system corresponding to Fig. 4d is a 2D SSH lattice with one out of four lattice sites in *all* unit cells excited, i.e., its on-site refractive index is changed in comparison to the other three sites in the unit cell. Even though this is a specific nonlinear excitation, it serves well to quantify how the topological feature is preserved after nonlinearity is introduced.

It is well known that, for the linear 2D SSH lattice, the polarizations are topologically quantized, $P_i = \frac{1}{2}$ for *c* < 0, and *P*_*i*_ = 0 otherwise. In the nonlinear case, the symmetry and topological protection are, strictly speaking, broken. However, we easily see from the illustrations that there is a sharp jump in the polarization as *c* crosses zero, which is inherited from the topological phase transition occurring in the underlying linear system. We see that the jump indicating this phase transition is preserved in the nonlinear system as well. We expect that such inherited nonlinear topological properties exist also in HOTIs of the third- or even higher-order formed in synthetic dimensions.

Finally, the ongoing debate about the use of breathing Kagome lattices for illustrating HOTI states merits further discussion, especially with respect to the recent work in ref. ^63^. First, our current work focuses on the topological BICs and their tunability by nonlinearity in a 2D SSH lattice, but such states do not exist in the Kagome lattice studied in ref. ^63^, which clearly distinguishes the two independent studies^51,52^. Second, there is a fundamental difference between chiral-symmetric topological crystalline insulators (such as the 2D SSH structure used in this work) that have a *C*_4*v*_ symmetry and those that do not have such an even-fold rotational symmetry^70,71^ (such as the Kagome lattices). As demonstrated recently, even though chiral symmetry is insufficient, on its own, to stabilize corner modes against strong perturbations, the additional presence of the fourfold rotational symmetry exhibited in the 2D SSH lattices does offer topological protection and further entails the formation of the topological BICs^54,55^. Moreover, it has been argued that the observed corner states in the breathing Kagome lattices^20,23,24^ may not be a manifestation of the HOTI characteristics^70,71^ as they simply lack for complete symmetry protection. Hence, our work on the nonlinear control of BIC-type corner states differs from those based on the breathing Kagome lattices. Such comparison and argument will stimulate further interest in topological photonics - one of the burgeoning photonic frontier areas^72^.

In conclusion, we have reported what we believe to be the first theoretical and experimental study of the nonlinear control of topological BICs in HOTIs. Understanding the nonlinear topological phases is not only of fundamental interest but it may also be crucial for the development of photonic devices based on topological corner modes, including HOTI lasers.

## Materials and methods

### Experimental methods for lattice writing and probing

To demonstrate the scheme for the nonlinear control illustrated in Fig. 1a, b, we employ a simple yet effective CW-laser writing technique^69^ to establish the desired finite-sized photonic 2D SSH lattices shown in Fig. 3a1–c1. The technique relies on writing the waveguides site by site in a 20-mm-long nonlinear photorefractive crystal (SBN:61 with cerium doping—0.002% CeO_2_). Different from the femtosecond laser writing method developed for glass materials^5^, the SSH lattices written in the crystal can be readily reconfigurable in terms of lattice spacing and boundary structures. The experimental set-up involves a low-power (up to 100 mW) laser beam (*λ* = 532 nm) to illuminate a spatial light modulator, which creates a quasi-non-diffracting writing beam with variable input positions. For the writing process, we use the modulated light beam (ordinarily polarized) with a self-focusing nonlinearity, but for probing during the nonlinear control process, we employ instead an extraordinarily polarized Gaussian beam for lattice excitation with either a self-focusing or self-defocusing nonlinearity, implemented conveniently by switching the bias field direction^64,65^. Because of the noninstantaneous photorefractive “memory” effect, all waveguides remain intact during the writing and the subsequently probing processes. (For the specific SBN crystal we used, the written index structure preserves for several hours in the dark or in the presence of only weak background illumination, but it can be readily erased with high-intensity white light and overwritten into another structure as needed.) Through a multi-step writing approach, the SSH lattice can be reconfigured from a nontrivial to a trivial structure by controlling the lattice spacing between the strong and weak bonds^67^. After the writing process is completed, the whole lattice structure can be examined by sending a Gaussian beam into the crystal to probe the waveguides one by one, and then the superimposed outputs of the probe beam display the lattice structure as shown in Fig. 3a1–c1. We note that the written waveguides are all single mode since a multi-mode waveguide by optical induction typically requires the formation of spatial solitons in a highly nonlinear regime^73^. It should also be pointed out that, differently from the conventional method of multi-beam induction in a biased crystal^64,65^, here the lattices are written by a CW-laser beam all with a positive bias field^69^, so for both trivial and nontrivial lattices, the index changes in all lattice sites follow the intensity distribution of the same writing beam (i.e., no “backbone lattice”^64,65^). After the writing process is completed, the probe beam is used to excite the lattice, and it can undergo either linear propagation (when the bias is turned off) or experience a self-focusing (or self-defocusing) nonlinearity under a positive (or negative) bias field. Of course, the probe beam can locally change the index structure of the lattices due to its self-action during nonlinear propagation—the ingredient needed for the nonlinear control.

### Numerical methods for beam propagation simulation and polarization calculation

The evolution of a light beam propagating in a photonic lattice is obtained by numerically solving Eq. (1) with the split-step Fourier technique, also referred to as the beam propagation method (BPM). The 2D SSH lattice structure has four lattice sites per unit cell (Fig. 1b), that is,

3 $$I_{\rm{L}}\left( {x,y} \right) = \mathop {\sum }\limits_{s = 1}^4 \mathop {\sum }\limits_{i = 0}^{N/4 - 1} \mathop {\sum }\limits_{j = 0}^{N/4 - 1} I_{{\rm{L}}0}\exp \left( { - \frac{{\left( {x - a_{sij}} \right)^2}}{{w_0^2/2}} - \frac{{\left( {y - b_{sij}} \right)^2}}{{w_0^2/2}}} \right)$$

where (*a*_1*ij*_, *b*_1*ij*_) = (*iT*, *jT*), (*a*_2*ij*_, *b*_2*ij*_) = (*a* + *iT*, *jT*), (*a*_3ij_, *b*_3*ij*_) = (*iT*, *a* + *jT*), and (*a*_4*ij*_, *b*_4*ij*_) = (*a* + *iT*, *a* + *jT*), with *T* = *a* + *b* being the lattice period, and *a* and *b* being the spacing between lattice sites for the weak and strong bonds (corresponding to intracell and intercell coupling in Fig. 1b, respectively). The total number of unit cells is *N*^2^/4, where *w*_0_ is a scaling factor and *I*_L0_ is the lattice magnitude. In the experiments, depending on the relative values between *a* and *b*, the photonic lattice can be reconfigured into a simple square lattice (*a* = *T*/2), a nontrivial SSH lattice for *a* > *T*/2 and a trivial SSH lattice for *a* < *T*/2. Similarly, we numerically excite only one corner (the left one in Fig. 3) with a Gaussian beam and perform BPM simulations for subsequent dynamics under linear and nonlinear conditions. Linear propagation results obtained with different lattice parameters are illustrated in Fig. 3a3–c3. For the nonlinear regime (*I*_P_ ≠ 0), simulations are performed for both self-focusing and self-defocusing nonlinearities in the nontrivial SSH lattice, and the results obtained at low and high nonlinearity are in good agreement with experimental observations (see Supplementary Material).

To characterize the topological properties of the 2D SSH lattices, we calculate the topological invariant based on the 2D polarization, which is defined for an infinite periodic system as^18,19^.

4 $$P_i = - \frac{1}{{\left( {2\pi } \right)^2}}{\int\!\!\!\!\!\int} {{\rm{d}}k_x{\rm{d}}k_yTr\left[ {A_i\left( {k_x,k_y} \right)} \right]}$$

where *i* = *x*, *y*, (*A*_*i*_)_*mn*_(**k**) = *i*〈*u*_*m*_(**k**)|$\partial_{k_i}$|*u*_*n*_(**k**)〉 is the Berry connection, and *u*_*m*_(**k**) is the eigenmode in the *m*th band. The 2D polarization is directly related to the 2D Zak phase: *Z*_*i*_ = 2*πP*_*i*_. One can readily calculate the polarization in the linear regime, which yields *P*_*x*_ = *P*_*y*_ = 1/2 for *c* < 0, and *P*_*x*_ = *P*_*y*_ = 0 for *c* > 0.

In order to test whether the signature of the topological phase transition at *c* = 0 is still present in the nonlinear regime, we calculate the *nonlinear* polarization by employing Eq. (4) for the following modified Hamiltonian applied to the 2D SSH lattices:

5 $$\displaystyle\hat H = \left( {\begin{array}{*{20}{c}} {\begin{array}{*{20}{c}} {\gamma _k} & {t + t^{\prime}\exp ( - ik_x)} \\ {t + t^{\prime}\exp (ik_x)} & 0 \end{array}} & {\begin{array}{*{20}{c}} {t + t^{\prime}\exp ( - ik_y)} & 0 \\ 0 & {t + t^{\prime}\exp ( - ik_y)} \end{array}} \\ {\begin{array}{*{20}{c}} {t + t^{\prime}\exp (ik_y)} & 0 \\ 0 & {t + t^{\prime}\exp (ik_y)} \end{array}} & {\begin{array}{*{20}{c}} 0 & {t + t^{\prime}\exp ( - ik_x)} \\ {t + t^{\prime}\exp (ik_x)} & 0 \end{array}} \end{array}} \right)$$

where *γ*_*k*_ accounts for the nonlinearity strength, and its sign manifests the difference between self-focusing and self-defocusing nonlinearities. This Hamiltonian corresponds to exciting one out of four lattice sites in all unit cells and changing its on-site potential via the employed nonlinearity. Calculated results for the nonlinear polarization are plotted in Fig. 4d, as a function of the dimerization parameter *c* = *t* − *t*′ defined earlier.

## Supplementary information

Suppl Mater

Movie 1

Movie 2
