# Supplementary material for: Nonlinear control of photonic higher-order topological bound states in the continuum
Source: Light Sci Appl. 2021 Aug 10;10:164. doi: 10.1038/s41377-021-00607-5 (PMC8355333; doi:10.1038/s41377-021-00607-5)
Supplement: Supplementary file 1 — Suppl Mater [file 41377_2021_607_MOESM1_ESM.pdf]

Supplementary Information for

Nonlinear control of photonic higher-order topological bound states in the continuum

Zhichan Hu[1†], Domenico Bongiovanni[1,2†], Dario Jukić[3], Ema Jajtić[4], Shiqi Xia[1], Daohong Song[1,5], Jingjun Xu[1,5], Roberto Morandotti[2,6], Hrvoje Buljan[1,4*], and Zhigang Chen[1,5,7*]

[1]The MOE Key Laboratory of Weak-Light Nonlinear Photonics, TEDA Applied Physics Institute and School of Physics, Nankai University, Tianjin 300457, China

[2]INRS-EMT, 1650 Blvd. Lionel-Boulet, Varennes, Quebec J3X 1S2, Canada

[3]Faculty of Civil Engineering, University of Zagreb, A. Kačića Miošića 26, 10000 Zagreb, Croatia

[4]Department of Physics, Faculty of Science, University of Zagreb, Bijenička c. 32, 10000 Zagreb, Croatia

[5]Collaborative Innovation Center of Extreme Optics, Shanxi University, Taiyuan, Shanxi 030006, China

[6]Institute of Fundamental and Frontier Sciences, University of Electronic Science and Technology of China, Chengdu, Sichuan 610054, China

[7]Department of Physics and Astronomy, San Francisco State University, San Francisco, California 94132, USA

[†]These authors contributed equally to this work

**hbuljan@phy.hr; *zgchen@nankai.edu.cn*

1. Animation of the eigenvalue evolution under nonlinear action

To substantiate the relevant discussion in the main text about the nonlinearity-induced beating oscillation between the corner and edge modes in higher-order topological insulators (HOTIs), under low self-focusing and -defocusing nonlinearities, we provide two AVI videos as supplementary material to show the full nonlinear band structure dynamics. Movie 1 is for a low *self-focusing* nonlinearity, where the four corner states (red spots) “dance” together and undergo periodic “touching” with the *lower* miniband to couple with the edge modes. Likewise, Movie 2 is for a low *self-defocusing* nonlinearity, where the four corner states (red spots) do the same and undergo periodic “touching” with the *upper* miniband of the edge modes.

2. Calculation of the band structure and nonlinear tuning of HOTI under a self-defocusing nonlinearity - the discrete model

In this section, we discuss nonlinear dynamics for the nontrivial 2D SSH lattice in the presence of a saturable self-defocusing nonlinearity, complementary to the self-focusing case whose results are presented in the main text of this manuscript. Fig. S1(a) illustrates the calculated nonlinear eigenvalue spectrum, superimposed on the corresponding linear band structure (see transparent dots) for direct comparisons. Numerical simulations are performed via the discrete NLSE model of Eq. (2) in the main text. In the *linear* regime (E_0' = 0), the nontrivial band structure is stationary (i.e., independent of the dimensionless propagation distance Z) and exhibits three main bands associated with extended bulk modes, plus two sets of edge modes residing in the mini-gaps. Additionally, it also displays four corner modes with zero eigenvalues (see red dots in Fig. S1(a)), which are embedded in the middle continuum bulk band. For this reason, they are topological bound states in the continuum (BICs), as discussed in the main text. Without loss of generality, the leftmost corner mode of the linear band structure is excited initially for testing the effect of nonlinearity. In the *nonlinear* regime ($E_0' \neq 0$), the spectrum evolves dynamically during propagation due to the action of the defocusing nonlinearity. The edge modes remain close to their linear positions. In contradistinction, the nonlinear corner modes move away from the middle band by experiencing an upward shift, eventually leading to interaction and beating with the upper-band edge modes at a low nonlinearity. As an example, a snapshot of the nonlinear spectral evolution is retrieved at the distance Z=50 for a low self-defocusing nonlinearity ($E_0' = -5, \gamma = 1.1$), showing

the whole spectrum up-shifted from its linear position and the corner modes approaching the upper edge modes. The beating and the energy exchange taking place between the edge and corner modes can be unveiled by looking at the transversal and longitudinal intensity distributions of the output beam (see Figs. S1(b2, b4)), which significantly deviates from the stable output profile of the corresponding linear corner mode (Fig. S1(b1)). Counterintuitively, the robustness of these corner-localized BICs is also preserved for the defocusing nonlinearity: similar to the self-focusing case, they do not couple with the bulk modes when moving in and out of the central bulk band.

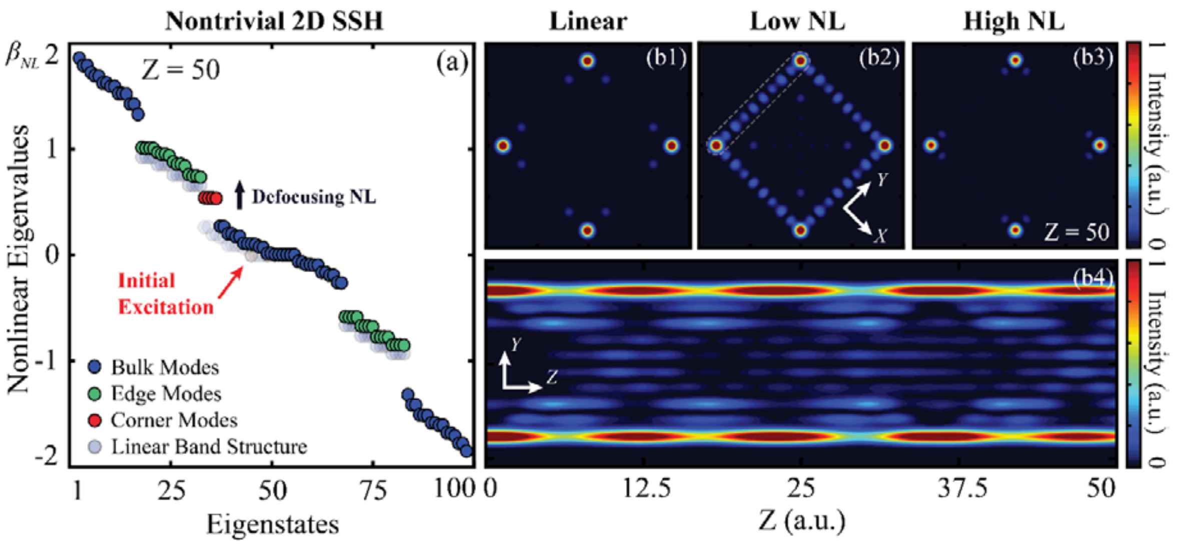

Figure S1. Calculated nonlinear band structure and corner mode tuning under a self-defocusing nonlinearity. (a) Calculated nonlinear eigenvalues β_{NL} for the nontrivial 2D SSH lattice using the discrete model taken at $Z = 50$; the transparent dots are linear mode eigenvalues plotted here for direct comparison. The black arrow points out that four corner states (red dots) undergo coupling and beating with the upper edge states under a low nonlinearity. The red arrow marks the initially excited corner mode, which possesses the topological features in the linear case, without any light distribution in the nearest neighboring sites as shown in (b1). Under a low self-defocusing nonlinearity, the corner mode couples with the edge modes (b2) so beating oscillations occur, as can be clearly seen from the side-view propagation in (b4), where we plot the intensity profile of the upper-left edge marked by a dashed line in (b2). Under a high defocusing nonlinearity, a corner-localized soliton-like pattern emerges (b3), which is quite different from the linear topological corner state shown in (b1). This can be inferred by looking at the nearest neighboring sites: the localization in this case is driven by nonlinearity, rather than topology.

For a sufficiently high defocusing nonlinearity ($E_0' = -5, \gamma = 3.5$), the corner modes are driven away from the continuum band, forming soliton-like nonlinear mode patterns, as calculated

from the discrete model. A typical example is shown in Fig. S1(b3). These results calculated using the discrete model under a self-defocusing nonlinearity are nearly identical to those obtained under a self-focusing nonlinearity (presented in Fig. 2 of the main text), which might be related to the chiral symmetry of the underlying linear system. However, it is worth mentioning that, in general, the results obtained from the discrete and the continuous models tend to deviate in the highly nonlinear defocusing regime as discussed in the main text.

We note that here the convention we used for calculating the eigenvalues is such that self-focusing pushes the eigenvalues downwards (see Fig. 2 of the main text), whereas defocusing moves them upwards (Fig. S1), to keep the analogy with condensed matter systems. In optics, this nonlinearity-driven up and down shifting directions could be reversed depending on how we define the propagation constant and plot the eigenvalue spectrum. More specifically, Eq. (2) from the main text reads $i\partial\psi/\partial Z + H_{NL}\psi = 0$. By using the condensed matter sign convention in the phase exponent $\psi = \varphi_{NL,n} exp\,(-i\beta_{NL,n}Z)$, we obtain $H_{NL}\varphi_{NL,n} = -\beta_{NL,n}\varphi_{NL,n}$. Because H_{NL} is Z-dependent, so are $\beta_{NL,n}$ and $\varphi_{NL,n}$. The convention used here does not change the physical content or any conclusions of the manuscript.

3. Simulation of nonlinear tuning of HOTI corner states - the continuous model

To further corroborate the experimental observations related to nonlinear control of the HOTIs presented in Fig. 4 of the main text, we perform numerical simulations via the split-step Fourier transform method, applied to the continuous paraxial nonlinear Schrödinger-like equation (NLSE):

$$i\frac{\partial\psi}{\partial z} + \frac{1}{2k}\left(\frac{\partial^2\psi}{\partial x^2} + \frac{\partial^2\psi}{\partial y^2}\right) - \Delta n\frac{\psi}{1+I_L+I_P} = 0 \qquad \text{(S1)}$$

where the periodic potential is included in the nonlinear term. Equation (S1) is the same as Eq. (1) in the main text, where $\psi(x, y, z)$ denotes the electric field envelope of the probe beam, whose intensity is I_P, and $I_L(x, y)$ represents the intensity of the linear 2D SSH lattice. Here x and y are the transversal coordinates, while z is the propagation distance. Furthermore, k is the wavenumber of the light in the medium, while Δn is the linear refractive index change determined by the bias field E_0 through $\Delta n = kn_e^2 r_{33}E_0/2$, where $n_e = 2.35$ and $r_{33} = 280$ pm V^{-1} are, respectively, the bulk refractive index and the electro-optics coefficient for extraordinarily polarized light in the photorefractive SBN:61 crystal. In principle, the overall index potential depends on the intensities

of both lattice-writing and probing beams, as well as the bias field E_0. Here we assume that the lattice index potential is set only by E_0, and simply let $I_P = \eta|\psi(x,y,z)|^2$ to control the nonlinearity induced by the probe beam. Therefore, for the linear case, $\eta = 0$, while for a positive (negative) value of η, the nonlinearity is self-focusing (-defocusing). This can properly simulate the experimental situation in the low saturation regime.

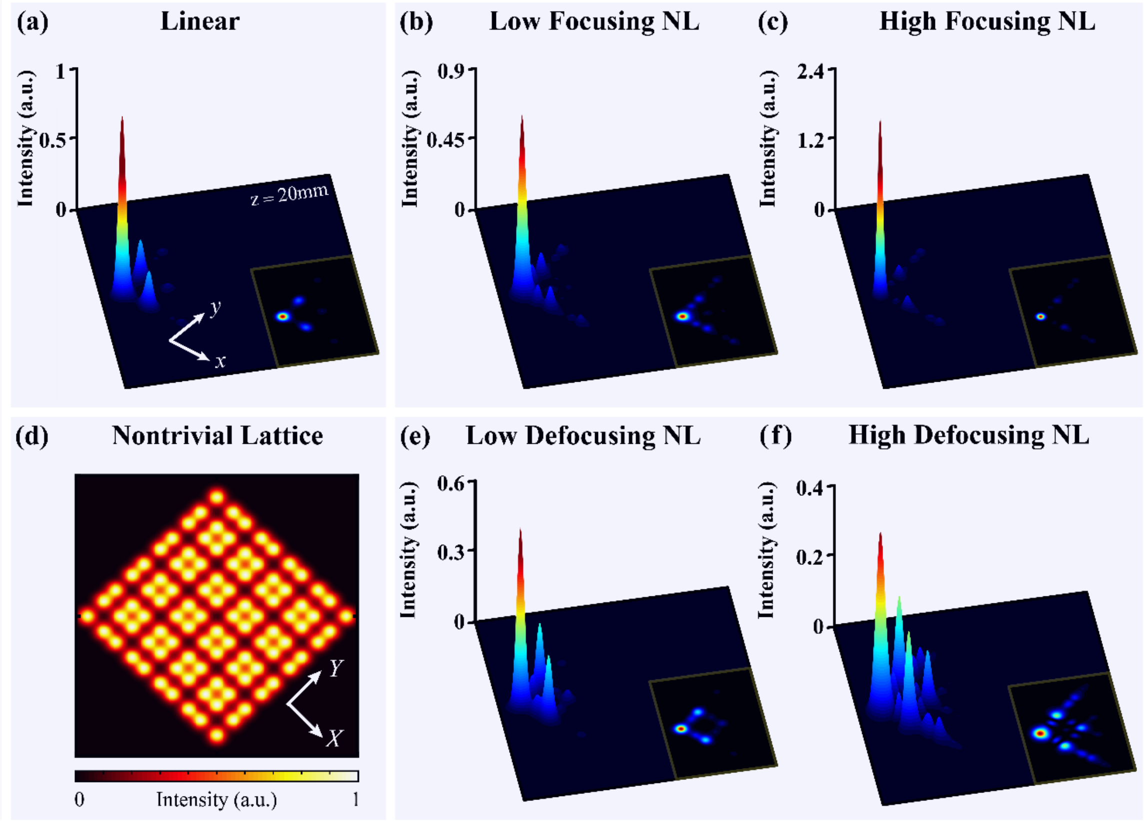

Figure S2. Numerical simulation of nonlinear control of a photonic higher-order topological insulator. (a) 3D-view of a linear corner state after 20 mm-long propagation through a nontrivial 2D SSH lattice. (b, c) Nonlinear *self-focusing* leads to (b) coupling of the corner mode to the edges (non-zero intensity in the edge sites compared with the linear case) when the nonlinearity is low, and (c) a highly localized corner soliton when the nonlinearity is high. (d) Transversal intensity distribution of the 2D SSH photonic lattice used in simulations. (e, f) Nonlinear output under a *self-defocusing* nonlinearity, corresponding to (b, c), showing strong energy spreading into both edge and bulk sites at a high nonlinearity strength. These results are in good agreement with the experimental results presented in Fig. 4 of the main text.

Numerical results for a 20 mm-long propagation (i.e., the crystal length used in the experiments) obtained from simulations with Eq. (S1) are presented in Fig. S2 for different

conditions, corresponding to the experimental results presented in Fig. 4 of the main text. Here, Fig. S2 also illustrates the output intensity profiles of the probe beam under a single corner excitation for a 2D SSH nontrivial lattice. Parameters in simulations are taken corresponding to our experimental setup. The 2D SSH lattice structure is defined with I_L in Eq. (3) of the main text. For the nontrivial lattice, the intra- and inter-cell distances are taken as $a = 31$ μm and $b = 23$ μm, the linear potential depth is $I_{L0} = 0.5$, and the scaling factor is $w_0 = 15.75$ μm. The intensity pattern of such a nontrivial lattice is illustrated in Fig. S2(d). The probe beam is a Gaussian profile with dimensions comparable to that of a lattice site. For simulations, we take $\Delta n = 2.36 \times 10^{-4} k/n_e$ for both the linear and defocusing cases and $\Delta n = 2.9 \times 10^{-4} k/n_e$ for the focusing case. Figure S2(a) illustrates the output probe beam with the characteristic features of the topological corner state under linear condition ($\eta = 0$). For low focusing ($\eta = 0.03$) and defocusing ($\eta = -0.05$) nonlinearities, the localized corner state differs from that of the topological linear system because the beam energy spreads in the nearest neighbor sites along two edges (see Figs. S2(b) and S2(e)). This is due to the nonlinearity-induced coupling between the corner and edge modes. However, at a high self-focusing nonlinearity ($\eta = 0.15$), the probe beam localizes again at the corner site of the lattice, but its intensity pattern does not have the features of a topological corner mode. In fact, it forms a self-trapped semi-infinite gap corner soliton (Fig. S2(c)). In contrast, at a high defocusing nonlinearity ($\eta = -0.65$), the corner excitation leads to strong spreading of the energy into both bulk and edges, as shown in Fig. S2(f). These numerical results are in good agreement with the experimental observations shown in Fig. 4 of the main text.

4. Characterization of corner BICs in logarithmic scale and their long-range propagation

In this section, we present the intensity plots of corner modes in both direct and logarithmic scales to substantiate our claims of topological BICs in the linear regime and their beating with the edge modes in the nonlinear regime. The example of the linear BIC profile shown in Fig. 1(d1) is performed using the discrete model, where there seems to be some background distribution inside the topological structure for the corner state. This is because the lattice structure is not large enough and as such, the “tails” of the corner modes seem to extend over the lattice structure. To show that this is not due to coupling with the bulk modes, that should not occur for true BICs, we recalculated the corner mode and plotted its profile in Figs. S3(a1-a3) with a doubled lattice size (20x20 sites).

The linear envelope in the logarithmic scale (Fig. S3(a3)) proves clearly its exponential localization. For the nonlinearity-induced beating oscillation described in Fig. 2 of the main text, we also performed the calculation for the nonlinear corner modes within a larger lattice. Snapshots are plotted in Fig. S3(b) (at Z = 23) and Fig. S3(c) (at Z = 50). At Z = 23, the corner mode couples with an edge mode (as seen from Movie 1), so as the intensity does not exhibit an exponential decay along the edges, yet there is no radiation into the bulk. At Z = 50, the corner mode tends to restore its topological BIC feature, as clearly seen from the intensity plots in Fig. S3(c3). This beating process goes back and forth periodically in the nonlinear regime, as described in the main text. It occurs for both self-focusing and -defocusing nonlinearities, but it cannot occur in the topological trivial structure. Strictly speaking, the nonlinear corner modes are no longer stationary BICs as in the linear regime, but they undergo periodic coupling with the edge modes without dissipating into the bulk, indicating the inherited topological nature of the corner states, even under nonlinear action.

In Fig. S4, we show numerical simulation results for long-distance propagation of the modes in the 2D SSH lattice using the continuous model. In the linear regime, the corner mode remains localized without coupling to the edge and bulk modes. To match our experimental condition, we launch a single beam into the corner waveguide, so we cannot say that the whole corner mode is excited, but rather a large portion of it. Clearly, even for long-distance propagation, most energy of the probe beam remains localized in the excited corner site. The weak oscillation of the intensity happens because we do not excite the exact corner mode, but rather one corner. In the right panel of Fig. S4, a weak self-focusing nonlinearity is applied, where the corner mode undergoes periodic intensity oscillation due to beating with the edge mode. The period of such beating oscillation is nearly 20 cm, much longer than the crystal length used in our experiments (which is only 20 mm). Interestingly, this kind of beating oscillation occurs even under a weak self-defocusing nonlinearity, although the defocusing nonlinearity speeds up the oscillation due to enhanced diffraction and coupling to the edge modes.

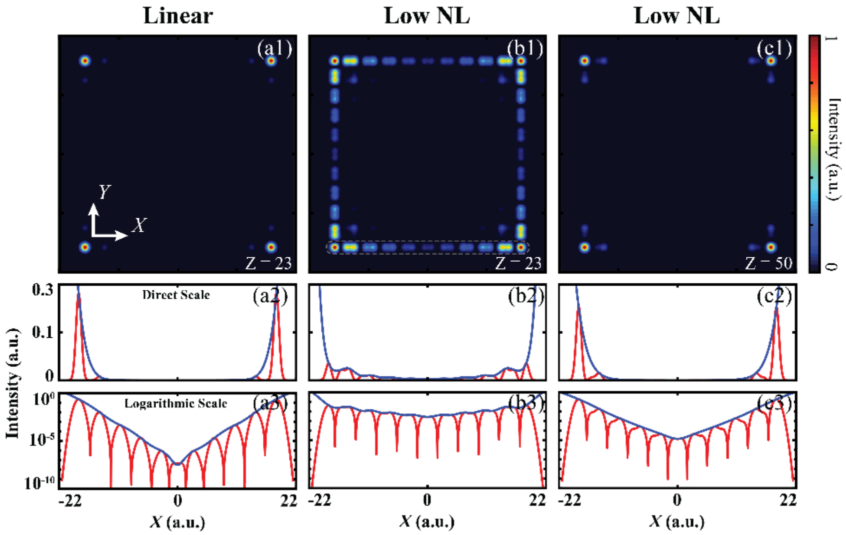

Figure S3. Intensity plots of a corner mode in both linear (a) and weakly nonlinear (b, c) regimes. The results were obtained using parameters corresponding to Fig. 1(d1) and Fig. 2(b2) in the main text, but with a double-sized 2D SSH lattice structure. In the nonlinear regime, the corner mode undergoes periodic oscillation between a mixed corner/edge state (b1) and an isolated corner state (c1), with no radiation into the bulk. Top panels are transverse intensity patterns, while the bottom panels are corresponding intensity profiles in direct and logarithmic scales. The linear envelope in the logarithmic scale proves clearly the exponential localization of the corner modes.

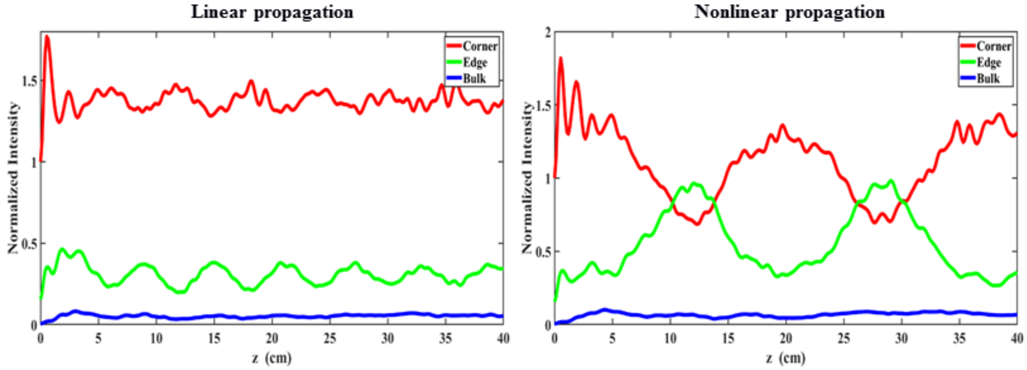

Figure S4. Numerical simulation of long-distance propagation of the modes in a photonic HOTI. Left: linear regime, where the corner mode remains localized without coupling with the edge and bulk modes. Right: weakly (self-focusing) nonlinear regime, where the average power in the corner sites undergoes a periodic oscillation due to beating with the edge mode, but not with the bulk mode.

5. Polarization as a function of nonlinearity

In this section, we discuss in more detail the nonlinear polarization which is presented in Fig. 4(d) of the main text. For linear systems the polarization of the 2D SSH model is quantized, such that $P_x = P_y = \frac{1}{2}$ in the topological nontrivial regime ($c < 0$), and $P_x = P_y = 0$ in the trivial regime ($c > 0$). As already elaborated in the main text, this simple picture is no longer valid when we introduce the nonlinearity γ_k, which breaks the symmetry protection of two distinct topological phases. However, we can still use polarization calculations to quantify the notion that the weakly nonlinear system can inherit topological properties from the linear system. More specifically, in Fig. S5 we plot the nonlinear polarization as a function of the nonlinearity γ_k for several values of the parameter c (which are chosen in both trivial and nontrivial regimes). We observe that the polarization slightly deviates from the linear case ($\gamma_k = 0$), i.e., from $P_x = P_y = 1/2$ when $c < 0$, and from $P_x = P_y = 0$ for $c > 0$. However, we also notice that the deviation is such that for weak nonlinearities (i.e., small $|\gamma_k|$) it grows only linearly with the nonlinearity parameter γ_k. In other words, if we imagine nonlinearity as a small perturbation of the corresponding linear system, we see that the characteristic topological features of the linear system (for example, a sudden jump in polarization) will be inherited in the nonlinear system as well.

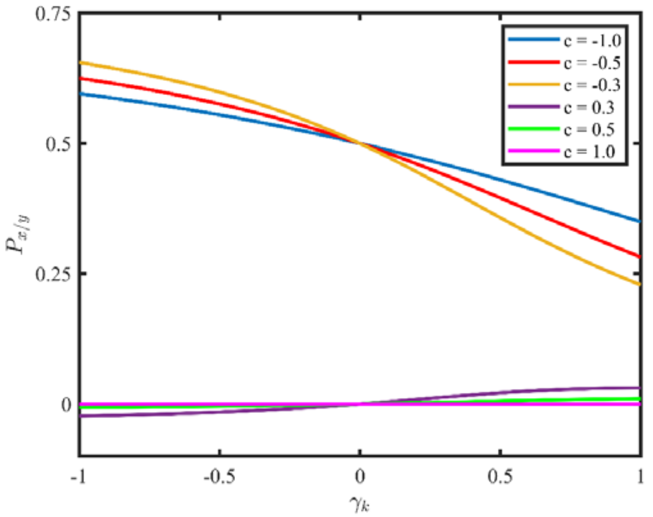

Figure S5. Polarization of the nonlinear 2D SSH system as a function of nonlinearity. The polarization $P_x = P_y$ of the nonlinear system depends on the strength of the nonlinearity parameter γ_k. For the linear systems ($\gamma_k = 0$), we know that $P_x = P_y = 1/2$ in the nontrivial regime ($c < 0$), and $P_x = P_y = 0$ in the trivial regime ($c > 0$). For weak nonlinearities, the deviation of the nonlinear polarization from this result grows linearly with the strength of the nonlinearity parameter γ_k.

Supplementary Movie 1: Dynamic eigenvalue evolution under low self-focusing nonlinearity.

Supplementary Movie 2: Dynamic eigenvalue evolution under low self-defocusing nonlinearity.
